# Supplementary material for: Impact of seasonal flooding and hydrological connectivity loss on microbial community dynamics in mangrove sediments of the southern Gulf of Mexico
Source: PeerJ. 2025 May 5;13:e19371. doi: 10.7717/peerj.19371 (PMC12060900; doi:10.7717/peerj.19371)
Supplement: Supplemental Information 1 [file peerj-13-19371-s001.docx]

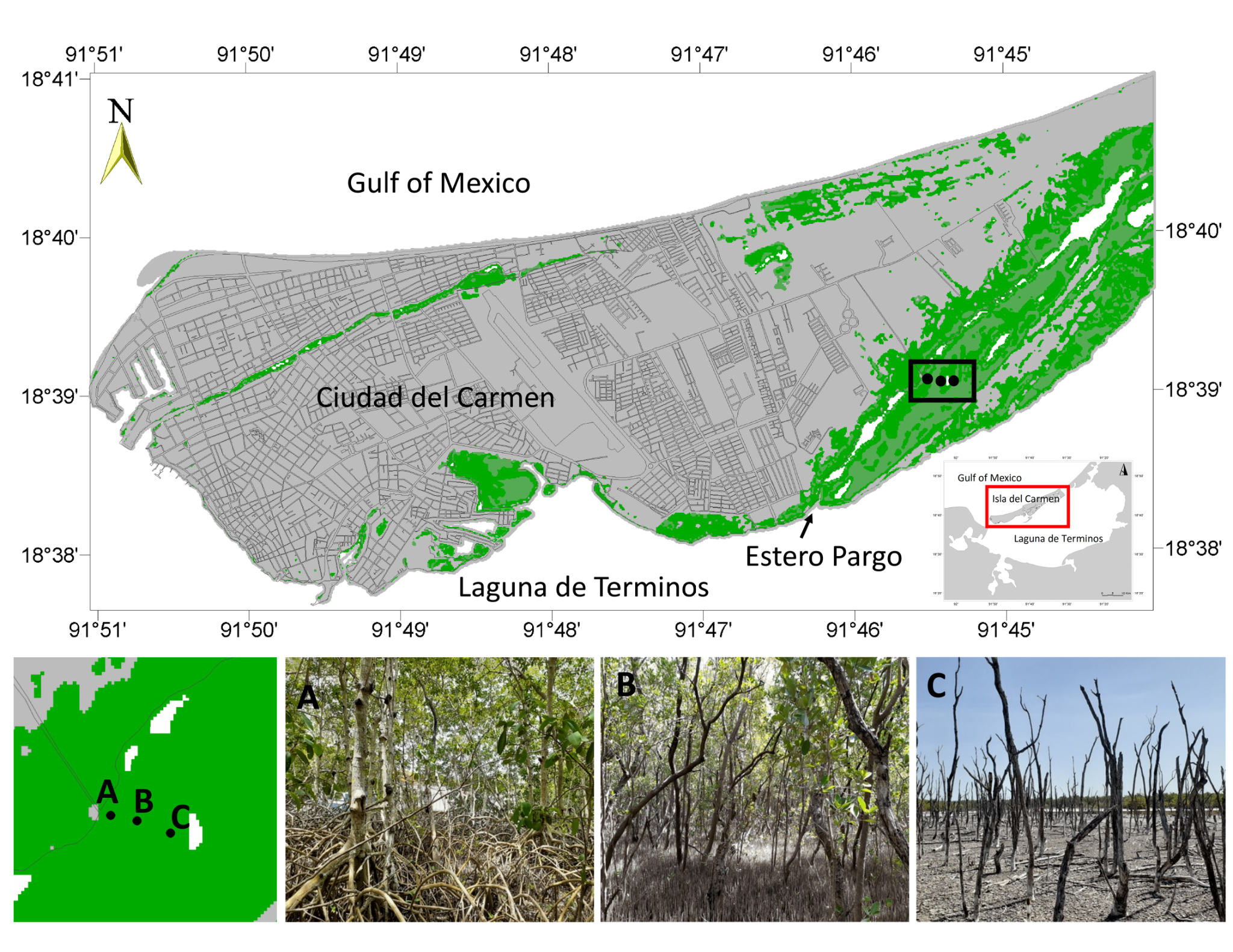


**Supplementary Figure 1:** Geographic and Ecological Overview of the Study Site. Panel A presents a broad geographic context with the location of Laguna de Términos and Estero Pargo, highlighting their proximity to the Gulf of Mexico and Ciudad del Carmen. Panel B details the specific sampling zones within Estero Pargo, identified as zones A (Fringe), B (Basin), and C (Impaired), each representing distinct environmental conditions: A (Fringe) features a pristine mangrove with dense vegetation; B (Basin) shows a mangrove under moderate environmental stress; C (Impaired) depicts a severely degraded mangrove with sparse vegetation and visible ecological stress. This figure provides a visual summary of the study's environmental settings, emphasizing the variation in mangrove health and structure across different zones within Estero Pargo.


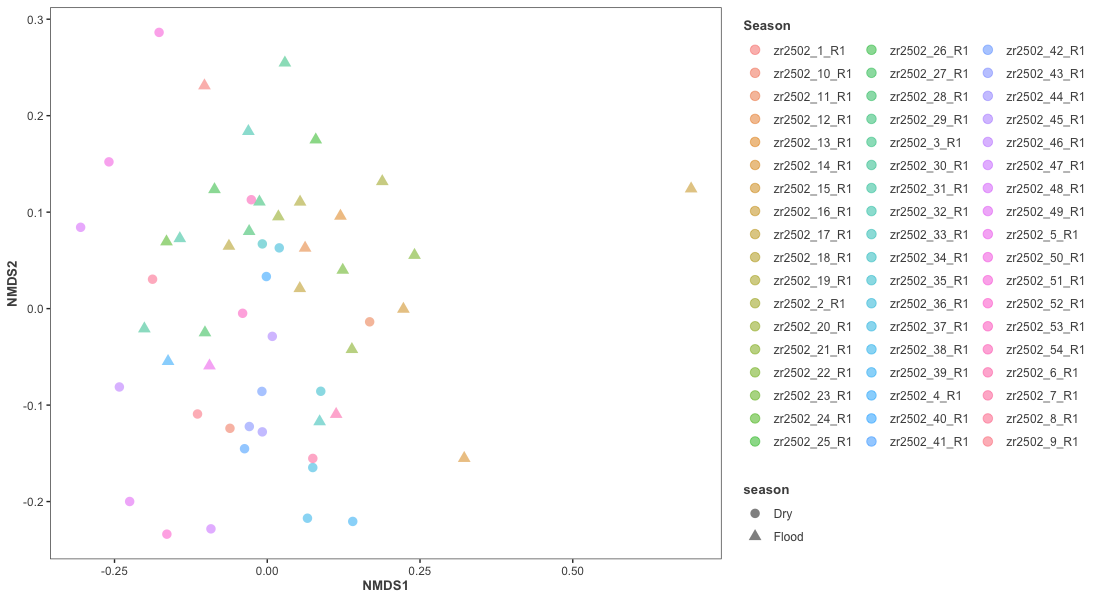


**Supplementary Figure 2: Non-metric multidimensional Scaling (NMDS) snalysis of amplicon sequence variants (ASVs).** This figure presents an NMDS plot of the microbial community composition, with each point representing a different sample, color-coded by sample and shaped by season. The plot highlights the relative similarity and differences in microbial communities across the samples. Notably, sample zr2502_16_R1 is positioned distinctly apart from the rest, indicating a rare microbial community composition relative to other samples.


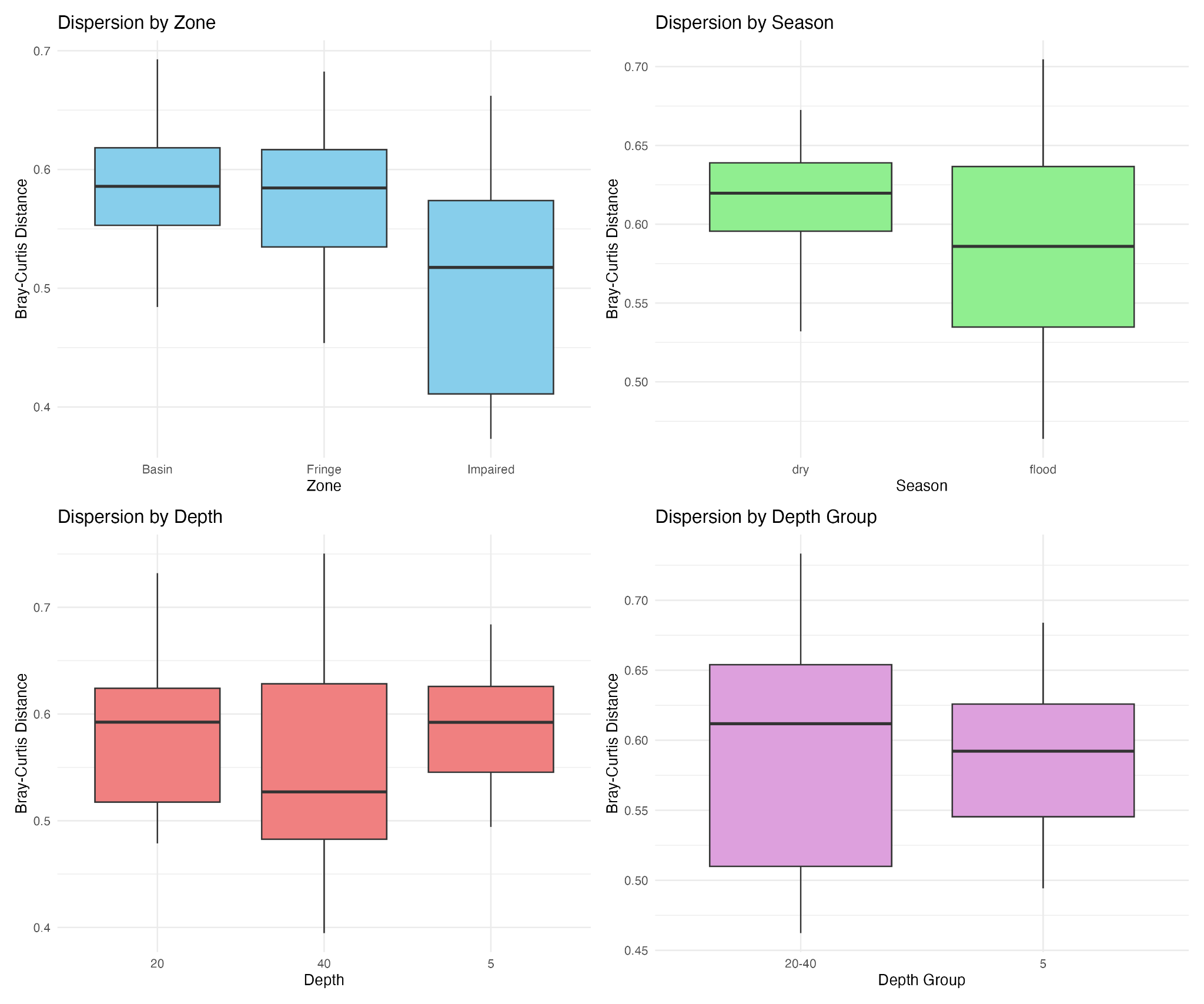


**Supplementary Figure 3: Beta diversity dispersion analysis using betadisper.** This figure displays the results of the Betadisper analysis, which assesses the homogeneity of multivariate dispersions within a dataset based on Bray-Curtis distances. Each plot represents the dispersion of data for different categorical variables tested: zone, season, and depth. These plots allow for the visual assessment of group dispersion, indicating the variability of microbial community composition within each category. This analysis helps to understand the influence of environmental factors such as geographic location, seasonal changes, and sediment depth on microbial diversity.
